# Arginase inhibition augments nitric oxide production and facilitates left ventricular systolic function in doxorubicin‐induced cardiomyopathy in mice

**DOI:** 10.14814/phy2.12130

**Published:** 2014-09-28

**Authors:** Takumi Toya, Daihiko Hakuno, Yasunaga Shiraishi, Takehiko Kujiraoka, Takeshi Adachi

**Affiliations:** 1 Division of Cardiology, Department of Internal Medicine, National Defense Medical College, Tokorozawa, Saitama, Japan; Takumi Toya, Kawasaki Municipal Hospital, 12‐1 ShinkawadooriKawasaki‐ku, Kawasaki, 210‐0013, Kanagawa, Japan; Daihiko Hakuno, Department of Cardiovascular Medicine, Kyoto University Hospital, 54 KawaharachoShogoin, Sakyo‐ku, 606‐8507, Kyoto, Japan

**Keywords:** Endothelial cell, heart failure, macrophage, nitric oxide

## Abstract

A metabolizing enzyme arginase can decrease nitric oxide (NO) production by competing with NO synthase for arginine as a substrate, but its pathophysiological role in heart failure remains unknown. We aimed to investigate the effect of pharmacological inhibition of arginase on left ventricular function in doxorubicin‐induced cardiomyopathy in mice. Doxorubicin administration for 5 weeks significantly increased protein expression levels or activity of arginase in the lungs and liver, and caused moderate increase in arginase 2 expression in the aorta. In the lungs, accumulated interstitial cells strongly expressed both arginase 1 and arginase 2 by doxorubicin administration. Echocardiography revealed that administration of a potent, reversible arginase inhibitor *N*‐omega‐hydroxy‐nor‐l‐arginine completely reversed doxorubicin‐induced decrease in the ejection fraction, in parallel with expression levels of BNP mRNA, without affecting apoptosis, hypertrophy, fibrosis, or macrophage infiltration in the left ventricle. Arginase inhibition reversibly lowered systolic blood pressure, and importantly, it recovered doxorubicin‐induced decline in NO concentration in the serum, lungs, and aorta. Furthermore, arginase inhibition stimulated NO secretion from aortic endothelial cells and peritoneal macrophages in vitro. In conclusion, pharmacological inhibition of arginase augmented NO concentration in the serum, lungs, and aorta, promoted NO‐mediated decrease in afterload for left ventricle, and facilitated left ventricular systolic function in doxorubicin‐induced cardiomyopathy in mice.

## Abstract (precis)

e12130

This figure shows that administration of an arginase inhibitor nor‐ NOHA facilitates LV systolic function in murine Dox‐induced cardiomyopathy.

## Introduction

Growing evidence suggests that nitric oxide (NO) is essential to the cardiovascular system in a cGMP‐dependent and ‐independent and redox state‐dependent manner (Schulz et al. 2005; Takimoto et al. 2005). In vascular systems, NO modulates vascular tonus, and reactive oxygen species (ROS) impairs NO bioactivity with the impaired endothelial NO synthase (eNOS), disrupts NO diffusion, and reduces NO response in smooth muscle cells (Luscher 1991; Tanner et al. 2000). In cardiomyocytes, NO/ROS balance also modulates cardiac systolic and diastolic function (Steppan et al. 2006; Heusch et al. 2010; Hammond and Balligand 2012). Thus, changes in the NO/ROS balance can be implicated in pathophysiology of heart failure (HF).

Arginase is an enzyme which metabolizes l‐arginine to form urea and l‐ornithine in urea cycle, and has two isoforms: cytosolic arginase 1 and mitochondrial arginase 2. Arginase 1 and 2 are mainly expressed in liver, and in kidney and intestine, respectively (Miyanaka et al. 1998), whereas both isoforms are expressed in endothelial cells (ECs), monocytes, and macrophages (Ming et al. 2012; Durante 2013). Arginase expression is upregulated by hypoxia (Krotova et al. 2010), shear stress, and oxidized low‐density lipoprotein (Ryoo et al. 2006) and downregulated by stimulation with estradiol in vitro (Hayashi et al. 2006). Besides l‐arginine metabolism, arginase can regulate NO production and redox state by competing with NOS for the common substrate l‐arginine, which causes further production of ROS with NOS uncoupling (Li et al. 2006). Therefore, increased arginase activity can be a prominent factor for the pathological imbalance between NO and ROS in cardiovascular systems.

Arginase 1 in alternatively activated macrophages exerts a beneficial effect by preventing excessive NO production by inducible NOS in acute inflammation (Ricardo et al. 2008). In contrast, deleterious effects of arginase have been vigorously studied, particularly in vascular diseases such as hypertension, atherosclerosis (Hayashi et al. 2006), and diabetes mellitus (DM) (Kashyap et al. 2008). *N*‐omega‐hydroxy‐nor‐l‐arginine (nor‐NOHA) is a synthetic analog of the intermediate product *N*‐omega‐hydroxy‐l‐arginine in NO production by NOS, and most potently and reversibly inhibits arginase activity without influencing NOS activity (Moali et al. 1998). Several recent reports have shown that nor‐NOHA administration effectively improves myocardial ischemia–reperfusion injury in rat (Jung et al. 2009) and EC dysfunction in hypertensive rat (Bagnost et al. 2010) and impaired flow‐mediated dilatation in patients with coronary artery disease and type 2 DM (Shemyakin et al. 2012).

Contrary to the aforementioned investigations, the pathophysiological role of arginase in the progression of HF remains largely unknown. A few reports revealed that arginase 2 protein levels are increased in murine mdx dystrophic heart (Wehling‐Henricks et al. 2010) and in pacing‐induced failing left ventricle (LV) of rabbits (Heusch et al. 2010), and circulating arginase 1 levels are increased in human HF (Quitter et al. 2013). However, the effect of arginase inhibition on LV function in HF remains unclear. We adopted doxorubicin (DOX)‐induced cardiomyopathy model, in which DOX causes overt LV systolic dysfunction due to dose‐dependent, progressive injury to cardiomyocytes mainly by ROS generation (Minotti et al. 2004).

The present study was designed to investigate whether pharmacological inhibition of arginase affects LV systolic function in DOX‐induced cardiomyopathy in mice.

## Methods

### Animal preparation

Male C57/BL6J mice were purchased from Clea Japan (Tokyo, Japan). Mice were kept under a 12‐h light–dark cycle and allowed ad libitum access to food and water. At 8 weeks of age, mice were randomly divided into three groups (*n* = 10 each): PBS, DOX, and DOX + NOHA groups. DOX (Sigma‐Aldrich, Tokyo, Japan) was intraperitoneally administered at a dose of 5 mg/kg once a week for 5 weeks in the DOX group. PBS group received an equal volume of PBS as a control. DOX + NOHA group was administered the equal dose of DOX and nor‐NOHA (Bachem, Bubendorf, Switzerland) at a dose of 40 mg/day intraperitoneally for 5 weeks. Mice were euthanized with intraperitoneal administration of pentobarbital and analyzed 8 weeks after commencement of each treatment.

Study protocol conformed to the Guide for the Care and Use of Laboratory Animals published by the United States National Institutes of Health and was approved by the ethics committee of animal research in our college (approval number 12034).

### Mouse echocardiography and blood pressure measurement

Mouse cardiac function at 8 weeks after commencement of treatment was assessed by the same blinded tester using echocardiography with a 45 MHz sector‐array transducer (Vevo 770; VisualSonics, Toronto, Canada). Mice were anesthetised with 1%–2% isoflurane inhalation during examination. Hearts were scanned at a rate of 100 frames/s using 2D or M‐mode. LV diameter and wall thickness were measured using M‐mode of the long‐axis view and averaged from 3–5 beats. Body weight and systolic blood pressure were serially measured till 8 weeks after commencement of treatment in conscious mice using the indirect tail‐cuff method for blood pressure (MK‐2000; Muromachi Kikai, Tokyo, Japan).

### Quantitative PCR

Total RNA was extracted from the LV using TRIzol reagent (Gibco, Carlsbad, CA). Purified RNA was dissolved in water, and first‐strand cDNA was synthesized using SuperScript III Reverse Transcriptase (Invitrogen, Carlsbad, CA), according to the manufacturer's instructions. Quantitative PCR was performed using Taqman Universal PCR Master Mix (Applied Biosystems, Carlsbad, CA) and FAM‐ or VIC‐labeled Taqman probes for BNP or GAPDH, respectively (Applied Biosystems). Samples were run in duplicates on the 7900HT Fast Real Time PCR System (Applied Biosystems). Relative expression levels were normalized to GAPDH (*n* = 5).

### Western blot analysis

Mouse tissues were homogenized in ice‐cold lysis buffer containing T‐PER (Thermo Scientific, Waltham, MA), 1 mmol/L sodium fluoride (Sigma‐Aldrich), 1 mmol/L sodium orthovanadate (Sigma‐Aldrich), complete protease inhibitor cocktail (Roche Applied Science, Penzberg, Germany) and 1 mmol/L phenylmethanesulfonyl fluoride (Sigma‐Aldrich). Samples were loaded onto 10% SDS gel, separated by electrophoresis, and transferred to PVDF membranes. Membranes were then incubated overnight with primary antibodies at 4°C. Primary antibodies used were as follows: anti‐arginase 1 (H‐52; Santa Cruz Biotechnology, Dallas, TX), anti‐arginase 2 (H‐64; Santa Cruz Biotechnology), anti‐β‐actin (4967; Cell Signaling Technology, Beverly, MA), anti‐caspase‐3 (H‐277; Santa Cruz Biotechnology), and anti‐GAPDH (MCA2427; AbD). After incubation with HRP‐conjugated secondary antibody and SuperSignal West Pico Chemiluminescent Substrate (Pierce, Waltham, MA), signals were visualized using LAS‐3000 mini (Fujifilm, Tokyo, Japan). The relative expression levels to β‐actin (*n* = 5) were quantified by the densitometric analysis using ImageJ 1.42q (National Institute of Health, U.S.A.).

### Histology, immunohistochemistry, and immunofluorescence staining

Mice were sacrificed and perfused with PBS and 4% paraformaldehyde: organs were immediately frozen in O.C.T. compound (Sakura Finetek Japan, Tokyo, Japan) or fixed overnight at 4°C and embedded in paraffin. Immunohistochemistry, immunofluorescence staining, and Masson's trichrome staining were performed as previously described (Hakuno et al. 2010). The following primary antibodies were used: anti‐arginase 1 (H‐52; Santa Cruz Biotechnology), anti‐arginase 2 (H‐64; Santa Cruz Biotechnology), anti‐α‐smooth muscle actin (SMA) (A5228, Sigma‐Aldrich), and anti‐Mac‐3 (BD Biosciences, San Jose, CA). Secondary antibodies for immunofluorescence staining were Alexa Fluor 546 and Alexa Fluor 488 goat IgG (Molecular Probes, Carlsbad, CA). The nuclei were stained with DAPI (Molecular Probes). The slides were observed under a microscope (AX80N‐65; OLYMPUS, Tokyo, Japan) or an immunofluorescence microscope (Biozero BZ‐8100; Keyence, Osaka, Japan). Cell size distribution of cardiomyocyte was assessed by measuring cross‐sectional area of 50 cardiomyocytes having nearly circular capillary profiles in the LV free wall. Histological images were analyzed by using ImageJ 1.42q.

### Arginase activity assay

Arginase activity was determined by measuring the amount of urea produced by a colorimetric method using QuantiChrom Arginase Assay Kit (BioAssay Systems, CA), according to the manufacturer's instructions. For tissue samples containing urea, urea was first depleted using a membrane filter (Amicon Ultra 10 kDa Ultracel; Millipore, Darmstadt, Germany).

### Apoptosis assay

Apoptosis in the murine LV was examined by TUNEL assay using In Situ Cell Death Detection Kit, Fluorescein (Roche Applied Science), according to the manufacturer's instructions. TUNEL‐positive cells were counted in six random fields for each LV under an immunofluorescence microscope.

### Oxidative stress measurement

Plasma concentration of hydroperoxide products was measured by the d‐ROMs test (FREE carpe diem; Diacron International, Grosseto, Italy) according to the manufacturer's instructions.

### NO metabolites measurement

NO metabolites concentration in serum and tissues was measured by the NO_2_/NO_3_ Assay kit‐FX (Fluorometric; DOJINDO, Kumamoto, Japan). Concentration of NO_2_^−^ and NO_3_^−^ was determined by the fluorometric method using NO_3_^−^ reductase and 2, 3–diaminonaphthalene, according to the manufacturer's instructions. For serum and tissue samples (*n* = 4), hemoglobin and proteins were removed using a membrane filter (Amicon Ultra 10 kDa Ultracel; Millipore) before initiation of the assay to prepare sample solutions. NO metabolite detection in the conditioned medium in vitro (*n* = 4), we used phenol red‐free medium followed by centrifugation at 1000 *g* for 15 min; the supernatant fraction was used as a sample.

### Cell preparation

Human aortic ECs were purchased from Takara Biotechnology, maintained as previously described (Hakuno et al. 2010), and used after 3–5 passages. Mouse peritoneal macrophages were harvested and cultured as previously described (Uto‐Kondo et al. 2013). To collect peritoneal macrophages, mice from the three groups were injected intraperitoneally with 10% thioglycollate (Difco, Detroit, MI). Three days later, macrophages were collected by lavage of the peritoneum with ice‐cold PBS, and the cells were seeded in 12‐well plates at a density of 3 × 10^6^ cells/well in DMEM with 10% FBS.

The cells were serum‐starved and stimulated with PBS, DOX (1 μmol/L) or DOX plus nor‐NOHA (100 μmol/L) for 12 h. Cells were then incubated with phenol red‐ and serum‐free medium for 2 or 48 h, and the conditioned media were collected for measurement of NO metabolites.

### Statistical analysis

All results are presented as mean ± SD. Statistical significance was evaluated using unpaired two‐tailed Student's *t* test for comparisons between two mean values. Multiple comparisons were performed using one‐way ANOVA with post‐hoc test by Tukey method. *P* values of < 0.05 were considered statistically significant.

## Results

### DOX administration augmented expression levels or activity of arginase in lungs and liver in mice

First, we investigated the changes in expression levels and activity of arginase isoforms by DOX administration in serum and various organs of mice. Previous studies showed that arginase 1 or 2 is mainly expressed in normal liver or in the kidney and intestine, respectively (Miyanaka et al. 1998). Moreover, both arginase isoforms are expressed in ECs and macrophages (Ming et al. 2012; Durante 2013).

**Figure 1.**
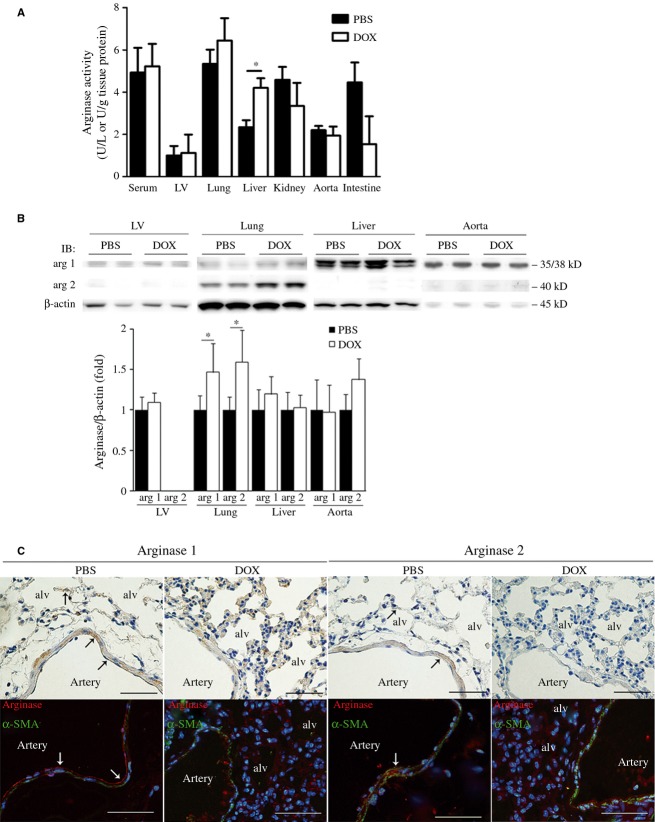
Doxorubicin administration augments expression levels or activity of arginase in lungs and liver in mice. Activity (A) and expression levels (B) of arginase isoforms in serum and various organs of mice in the PBS and DOX groups. Changes in expression levels were examined by western blot and densitometric analysis. The activity increased in lungs and liver in the DOX group, whereas the expression levels of both isoforms significantly augmented in lungs. There was a trend of increase (*P *=**0.10) in arginase 2 expression in the aorta. arg 1, arginase 1; arg 2, arginase 2. *P <**0.05 versus PBS. C. Localization of arginase isoforms in lungs. DAB staining (brown) and triple immunofluorescence staining (arginase, red; α‐SMA, green; nuclei, blue) are shown in the upper and lower rows, respectively. Arginase 1 was expressed in ECs and smooth muscle cells of pulmonary arteries and lung epithelial cells, whereas arginase 2 appeared mainly expressed in the smooth muscle cells and epithelial cells (arrows). DOX administration accumulated arginase‐expressing epithelial and interstitial cells. alv, alveoli; α‐SMA, α‐smooth muscle actin. Scale bars, 100 μm (DAB) and 50 μm (immunofluorescence).

Arginase activity was increased in the lungs and liver by DOX administration, with significant increase by 1.8 ± 0.3‐fold in the liver (Fig. 1A). In contrast, the activity was unchanged in serum, LV, and aorta, and was decreased in kidneys and intestine.

Western blot analysis found that protein expression levels of arginase 1 and 2 were significantly augmented in lungs, but not in liver by DOX administration (Fig. 1B). There was a trend of increase (*P *=**0.10) in arginase 2 expression in the aorta. Posttranslational modification might explain the discrepancy between arginase activity and expression levels in lungs by DOX administration. To further determine the localization of arginase isoforms in lungs, immunohistochemistry and immunofluorescence staining were performed with arginase isoform‐specific antibodies (Fig. 1C). We found that arginase 1 was expressed in ECs and smooth muscle cells of pulmonary arteries and lung epithelial cells, whereas arginase 2 appeared mainly expressed in smooth muscle cells and epithelial cells in the PBS group (arrows in Fig. 1C). Interestingly, the expression levels of both isoforms increased in accumulating epithelial and interstitial cells, but not in the ECs and smooth muscle cells by DOX administration.

Therefore, DOX administration augmented expression levels or activity of arginase in lungs and liver in mice.

### Arginase inhibition facilitated LV systolic function in DOX‐induced cardiomyopathy in mice

**Figure 2.**
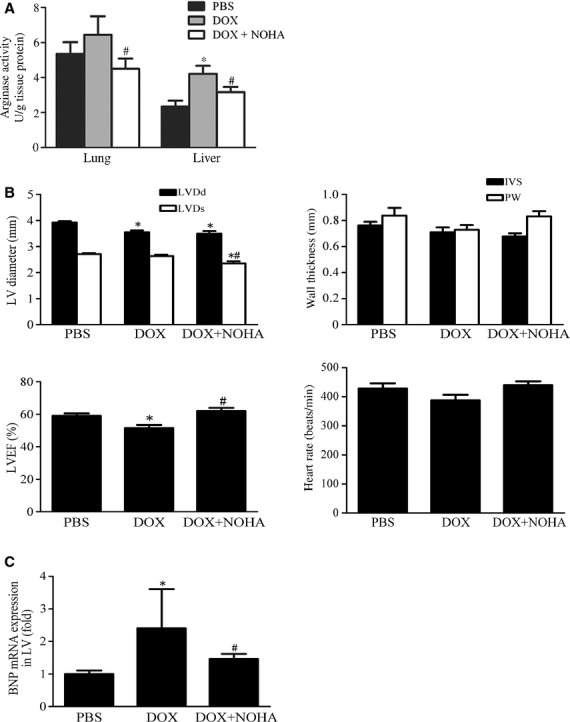
Arginase inhibition facilitates LV systolic function in DOX‐induced cardiomyopathy in mice. (A) Changes in arginase activity in lungs and liver of PBS, DOX, and DOX plus arginase inhibitor nor‐NOHA (DOX + NOHA) groups. Simultaneous administration of nor‐NOHA significantly decreased arginase activity in the DOX + NOHA group compared with the DOX group. *P <**0.05 versus PBS; ^#^*P *<**0.05 versus DOX. (B) Echocardiographic findings in the LV and heart rate of PBS, DOX, and DOX + NOHA groups. The LVEF significantly decreased in the DOX group compared with the PBS group, and this was almost completely reversed in the DOX + NOHA group. Heart rate was similar among the three groups. Dd, end‐diastolic diameter; Ds, end‐systolic diameter; IVS, interventricular septum; PW, posterior wall. (C) Expression levels of BNP mRNA examined using quantitative PCR. Levels increased in the LV of the DOX group compared with the PBS group, and significantly reduced in the DOX + NOHA group. *P <**0.05 versus PBS; ^#^*P *<**0.05 versus DOX.

To investigate whether arginase inhibition favors LV function in DOX‐induced cardiomyopathy in mice, PBS, DOX, or DOX plus a potent, reversible arginase inhibitor nor‐NOHA (DOX + NOHA) was repetitively administered. Simultaneous administration of nor‐NOHA significantly decreased arginase activity in lungs and liver of the DOX + NOHA group compared with the DOX group, confirming the pharmacological inhibition of arginase (Fig. 2A).

Echocardiography found that LV diameter at end‐diastole was decreased in the DOX and DOX + NOHA groups compared with the PBS group, but LV diameter at end‐systole was further reduced only in the DOX + NOHA group (Fig. 2B). There were no significant difference in wall thickness and heart rate among the three groups. However, LV ejection fraction significantly decreased in the DOX group compared with the PBS group (50.9 ± 2.9% vs. 59.3 ± 3.6%, respectively), whereas nor‐NOHA administration almost completely reversed DOX‐induced decline in LV ejection fraction (62.4 ± 2.9%, *P *<**0.05 vs. DOX group).

Expression levels of BNP mRNA increased by 2.5 ± 1.1‐fold in the LV of the DOX group compared with the PBS group; this increase reduced by 67% in the DOX + NOHA group (Fig. 2C, *P* < 0.05 vs. DOX group).

These data indicate that arginase inhibition facilitated LV systolic function in DOX‐induced cardiomyopathy in mice, presumably by increasing myocardial contractility or by decreasing preload and afterload for LV.

### Arginase inhibition did not affect DOX‐induced LV remodeling in mice

We then hypothesized two mechanisms for facilitation of LV systolic function by arginase inhibition: (1) LV remodeling alleviation; (2) increase in systemic NO concentration and blood pressure lowering.

**Figure 3.**
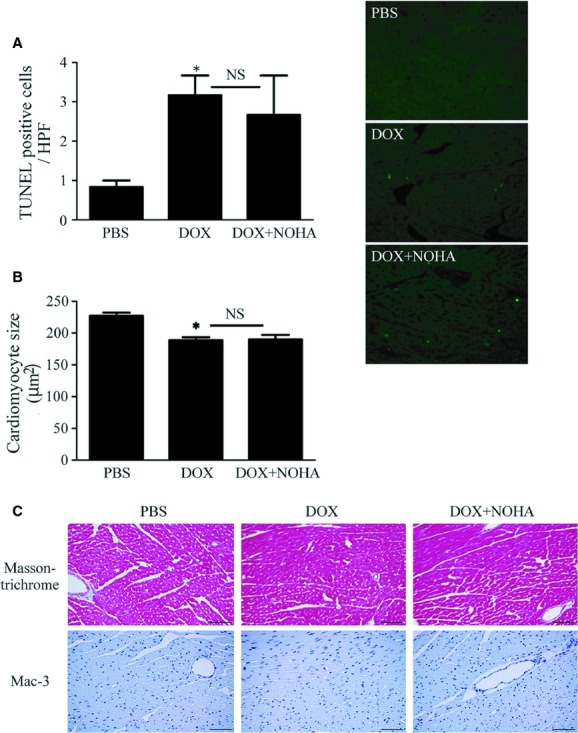
Arginase inhibition does not alleviate DOX‐induced LV remodeling in mice. (A) Cardiomyocyte apoptosis examined by the TUNEL assay in the LV of each group. Number of TUNEL‐positive cardiomyocytes (green in right images) significantly increased in the DOX group compared with the PBS group, which was unaffected by arginase inhibition. Scale bars, 200 *μ*m. *P <**0.05 versus PBS; NS, not significant. (B) Cardiomyocyte size of 50 cells per group was measured using Image J. Cardiomyocytes were smaller in the LV of DOX administered hearts, which was not affected by arginase inhibition. *P <**0.05 versus PBS; NS, not significant. (C) LV fibrosis assessed by Masson's trichrome staining. An extent of myocardial fibrosis was unchanged by arginase inhibition. Scale bars, 100 *μ*m. D. Macrophage infiltration detected by immunostaining with Mac‐3 was not obvious in the LV of any group. Inset shows splenocytes as a positive control. Scale bar, 100 *μ*m.

First, we analyzed the effect of arginase inhibition on LV remodeling with regard to myocardial apoptosis, fibrosis, and macrophage infiltration in mice. DOX administration significantly increased TUNEL‐positive cardiomyocytes (Fig. 3A) and decreased cardiomyocyte size (Fig. 3B), indicating increase in myocardial apoptosis and atrophy. However, arginase inhibition could not reverse DOX‐induced apoptosis and atrophy. Similarly, Masson's trichrome staining revealed that an extent of myocardial fibrosis was unchanged by arginase inhibition (Fig. 3C). Macrophage infiltration examined by immunostaining with Mac‐3 antibody was not obvious in any group (Fig. 3D).

These data indicate that arginase inhibition did not affect DOX‐induced LV remodeling in mice

### Chronic inhibition of arginase reversibly lowered tail blood pressure in DOX‐induced cardiomyopathy in mice

**Figure 4.**
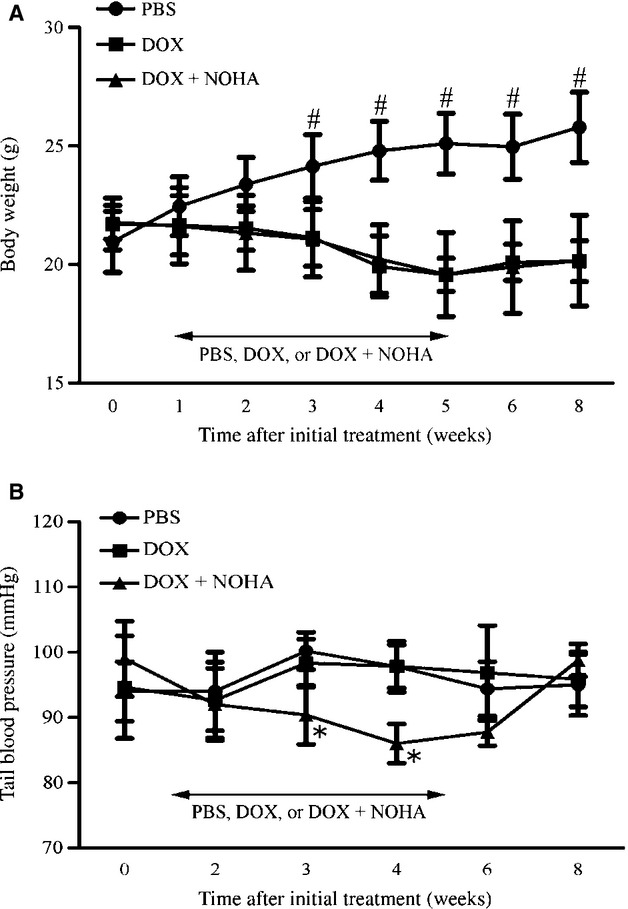
Arginase inhibition reversibly lowers tail blood pressure in DOX‐induced cardiomyopathy in mice. Serial changes in body weight (A) and systolic blood pressure (B) were measured. Body weight was decreased during DOX administration regardless of nor‐NOHA administration. Chronic inhibition of arginase lowered systolic blood pressure at 4 weeks after the initial administration compared with that in PBS and DOX groups, whereas blood pressure was gradually restored after stopping administration. ^#^*P *<**0.05 versus DOX and DOX + NOHA; *P <**0.05 versus PBS and DOX.

Next, we serially measured body weight and tail blood pressure to assess systemic and hemodynamic effects of DOX administration and arginase inhibition. Body weight was gradually decreased during DOX administration as compared to that in PBS with statistical difference after 3 weeks of initial administration regardless of nor‐NOHA administration (Fig. 4A). DOX administration did not change tail blood pressure throughout the study period as compared to PBS (Fig. 4B). In contrast, simultaneous arginase inhibition significantly lowered systolic blood pressure up to 12.1 ± 3.1% at 4 weeks after initial administration as compared with PBS and DOX (*P *<**0.05), and blood pressure gradually recovered after stopping nor‐NOHA administration. There was no significant difference in systolic blood pressure among the three groups at 8 weeks.

These findings demonstrate that pharmacological inhibition of arginase reversibly lowered blood pressure with resultant decrease in afterload for LV in DOX‐induced cardiomyopathy in mice.

### Arginase inhibition augmented NO concentration in serum, lungs, and aorta in DOX‐induced cardiomyopathy in mice

**Figure 5.**
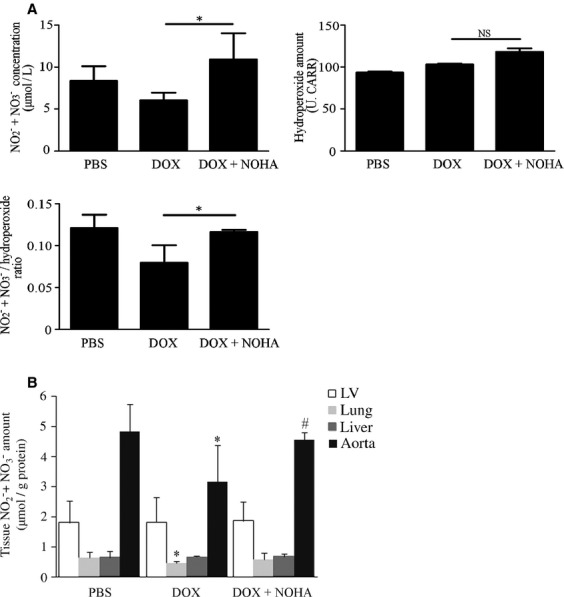
Arginase inhibition augments NO concentration in serum, lungs, and aorta in DOX‐induced cardiomyopathy in mice. NO concentration in serum (A) and several tissues (B) of each group. (A) NO concentration in serum decreased with DOX administration; the decline was markedly augmented in the DOX + NOHA group. Although the serum hydroperoxide level slightly increased in the DOX + NOHA group, DOX‐induced decrease in serum NO/ROS ratio significantly improved to the control level. *P <**0.05; NS, not significant. (B) NO concentration decreased significantly in the lungs and aorta by DOX administration, whereas this decline was reversed in the DOX + NOHA group. *P <**0.05 versus PBS; ^#^*P *<**0.05 versus DOX.

To examine whether arginase inhibition increases systemic NO concentration, we analyzed changes in NO concentration in serum and several organs by arginase inhibition. Serum NO concentration decreased with DOX administration, whereas this decline was markedly augmented by 116.4 ± 5.9% in the DOX + NOHA group (Fig. 5A, *P* < 0.05 vs. DOX group). Serum hydroperoxide level increased slightly, but not significantly in the DOX + NOHA group compared with the DOX group. Nevertheless, DOX‐induced decrease in serum NO/ROS ratio (denoted as NO_2_^−^ + NO_3_^−^ concentration divided by hydroperoxide level) significantly recovered to the control level in the DOX + NOHA group (*P *<**0.05 vs. DOX group).

Furthermore, we found that NO concentration significantly decreased to 70.9 ± 11.9% and 65.8 ± 24.7% in lungs and aorta, respectively, with DOX administration, whereas this decline was reversed in the DOX + NOHA group, with significant increase in aorta (Fig. 5B, *P* < 0.05 vs. DOX group). In contrast, NO concentration was unchanged in LV and liver in the three groups, in compatible with the previous report that NOS expression levels are low in the steady state of the liver (Suematsu et al. 1995).

These data indicate that arginase inhibition augmented NO concentration in serum, lungs, and aorta and improved the systemic balance between NO and ROS concentration in DOX‐induced cardiomyopathy in mice. These findings strongly suggest that arginase inhibition facilitated LV systolic function by NO‐mediated decrease in afterload for LV.

### Arginase inhibition stimulated NO secretion from aortic ECs in vitro and peritoneal macrophages *ex vivo*

**Figure 6.**
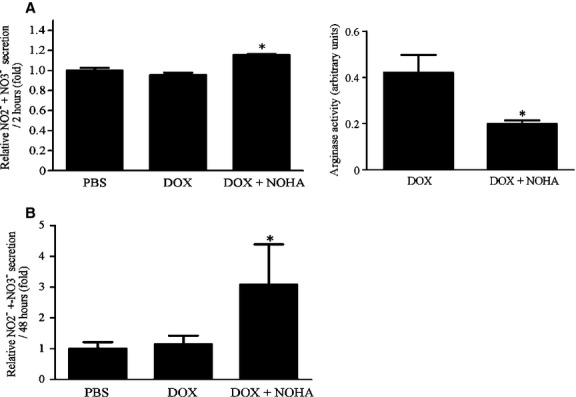
Short‐term inhibition of arginase stimulates NO secretion from ECs and macrophages in vitro. Short‐term effect of arginase inhibition on NO secretion from human aortic ECs in vitro (A, left) and mouse peritoneal macrophages *ex vivo* (B). Arginase activity was suppressed to less than half by nor‐NOHA in vitro experiments using ECs (A, right). NO concentration in the conditioned media of ECs and macrophages significantly increased in the DOX + NOHA group compared with the DOX group. *P <**0.05 versus DOX.

To further investigate the cellular targets of arginase inhibition in vivo, we performed in vitro experiment using human aortic ECs and *ex vivo* experiment using mouse peritoneal macrophages. Nor‐NOHA administration was sufficient to inhibit enzymatic activity of arginase to less than half in vitro experiments using ECs (Fig. 6A, right). NO secretion from ECs (Fig. 6A, left) and peritoneal macrophages (Fig. 6B) significantly increased by 20.8 ± 2.2% and 171.0 ± 103.3%, respectively, in the DOX + NOHA group compared with the DOX group (*P *<**0.05 vs. DOX group).

These findings indicate that even short‐term inhibition of arginase stimulated NO secretion from aortic ECs and macrophages in vitro, and that these cells may have been the main targets of arginase inhibition in vivo.

## Discussion

In the present study, we demonstrated that pharmacological inhibition of arginase augmented NO concentration in serum, lungs, and aorta, normalized serum NO/ROS ratio, and facilitated LV systolic function in DOX‐induced cardiomyopathy in mice.

Arginase inhibition could increase NO production by improving l‐arginine availability for NOS and could decrease ROS production by reducing NOS uncoupling. We found that DOX administration significantly increased activity or expression levels of both arginase 1 and arginase 2 in the liver and lungs. In addition, there was a trend of increase in arginase 2 expression in the aorta. The discrepancy between arginase activity and expression levels in lungs by DOX administration might be explained by posttranslational modification. Interestingly, accumulated interstitial cells, which would contain inflammatory cells, strongly expressed both arginase 1 and arginase 2 in the lungs by DOX administration, indicating the cause of increase in arginase expression. On the other hand, arginase inhibition by a potent arginase inhibitor nor‐NOHA reversibly decreased systolic blood pressure, and restored DOX‐induced decrease in NO concentration in serum, lungs, and aorta. Furthermore, in vitro study revealed that arginase inhibition significantly stimulated NO secretion from aortic ECs and macrophages. Therefore, we assumed that arginase inhibition targeted lungs and aorta, especially ECs and inflammatory cells including macrophages, and facilitated LV systolic function by NO‐mediated decrease in vascular tonus and afterload for LV in parallel with BNP reduction in this mouse model. Although arginase activity did not change after DOX administration in the aorta (Fig. 1A), NO concentration was significantly decreased. The discrepancy of these results can be explained by the fact that amount of ECs accounts for quite a little of aortic tissues compared to vascular smooth muscle cells.

Previous reports showed that acute or chronic inhibition of arginase with nor‐NOHA improved vascular function in spontaneously hypertensive rats (Bagnost et al. 2010) and in humans (Shemyakin et al. 2012) in an NO‐dependent manner. Furthermore, it was reported that long‐term dietary nitrate supplementation resulting in a substantial increase in plasma NO level protects against murine DOX‐induced cardiomyopathy by improving mitochondrial function and LV contractility (Zhu et al. 2011). The net balance between NO and ROS concentration is the primary determinant of cellular redox state. Therefore, it is reasonable that increase in NO production from extracardiac organs, particularly from ECs and macrophages, by arginase inhibition favors LV function in DOX‐induced cardiomyopathy. Although dysregulated arginine metabolism (Shao et al. 2012) and diminished arginine bioavailability (Tang et al. 2013) were noticed in patients with systolic HF, our study highlights the pathophysiological role of arginase and its possible therapeutic target in this disease.

With respect to possible arginase effects on LV contractility, Steppan et al. (2006) reported that inhibition of arginase 2 augmented the contractility of isolated cardiomyocytes. Moreover, NOS 1 enhanced cardiomyocyte contractility through an S‐nitrosylation mechanism by activating ryanodine receptors, which colocalized with NOS 1 in the cardiomyocytes, and that mitochondrial arginase 2 and NOS 1 in the sarcoplasmic reticulum formed a microdomain. Although arginase 2 protein was only slightly expressed in the LV of three groups in the present study, there was a possibility that nor‐NOHA improved LV contractility by inhibiting cardiac mitochondrial arginase 2. In addition, localization and expression levels of NOS isoforms might affect cardiac function by arginase inhibition.

Other considerable factors that may affect NO production levels include tetrahydrobiopterin (BH_4_), asymmetric dimethylarginine (ADMA), dimethylarginine dimethylaminohydrolase (DDAH), and cationic amino acid transporter (CAT). BH_4_ is a coenzyme of NOS, and its impairment leads to NOS uncoupling, resulting in decreased NO production and increased ROS generation (Antoniades et al. 2006). ADMA is a methylated arginine analog that works as an endogenous competitive inhibitor of all NOS isoforms. Serum ADMA concentration increases in a wide range of diseases including DM, and ADMA accumulation causes endothelial dysfunction by NOS inhibition (Hink et al. 2001). One of the major causes of ADMA accumulation is the disorder of DDAH activity that metabolizes ADMA (Dayoub et al. 2003; Torondel et al. 2010). Abnormal uptake of l‐arginine via CAT contributes to endothelial dysfunction in cardiovascular diseases. However, the involvement of these factors in NO production remained unclear in this study.

In conclusion, the present study demonstrated that arginase inhibition facilitated LV systolic function in DOX‐induced cardiomyopathy in mice, presumably by targeting lungs and aorta, especially ECs and macrophages and by promoting NO‐mediated decrease in afterload for LV. Further studies are needed to determine whether inhibition of arginase 1 or arginase 2 has more beneficial effect and can be applied to the broader spectrum of HF.

## Conflict of Interest

None.
